# Airborne Bacterial Communities: Diversity, Survival Strategies and Functional Roles in the Atmosphere

**DOI:** 10.1111/1758-2229.70274

**Published:** 2026-01-08

**Authors:** Jungsoo Park, S. Jane Fowler

**Affiliations:** ^1^ Department of Biological Sciences Simon Fraser University Burnaby Canada

## Abstract

The atmosphere is increasingly recognised as a dynamic microbial habitat, yet the mechanisms that enable bacterial survival in air remain underexplored. This mini‐review synthesises current knowledge on airborne bacterial diversity, the selective pressures they face and the traits that support survival. Drawing from environmental surveys, laboratory studies and emerging omics data, we highlight how airborne bacteria survive despite extreme conditions including UV radiation, low water activity, oxidative conditions and limited nutrients. Common traits such as DNA repair, pigmentation, antioxidant systems and spore formation are discussed in relation to atmospheric stress. We also review recent evidence of microbial activity and function in air. By integrating ecological patterns with physiological adaptations, this review outlines how specific traits may contribute to survival in the atmosphere and suggests future directions for functional studies in diverse atmospheric environments.

## Graphical abstract

This mini‐review synthesises evidence on the taxonomic patterns of airborne microorganisms and the selective pressures they face. We highlight key conserved physiological traits, such as DNA repair and desiccation tolerance, which enable stress‐tolerant members to survive and suggest their potential for activity within the atmospheric environment.

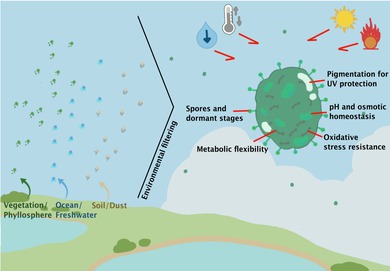

## 1 Introduction

**FIGURE 1:**
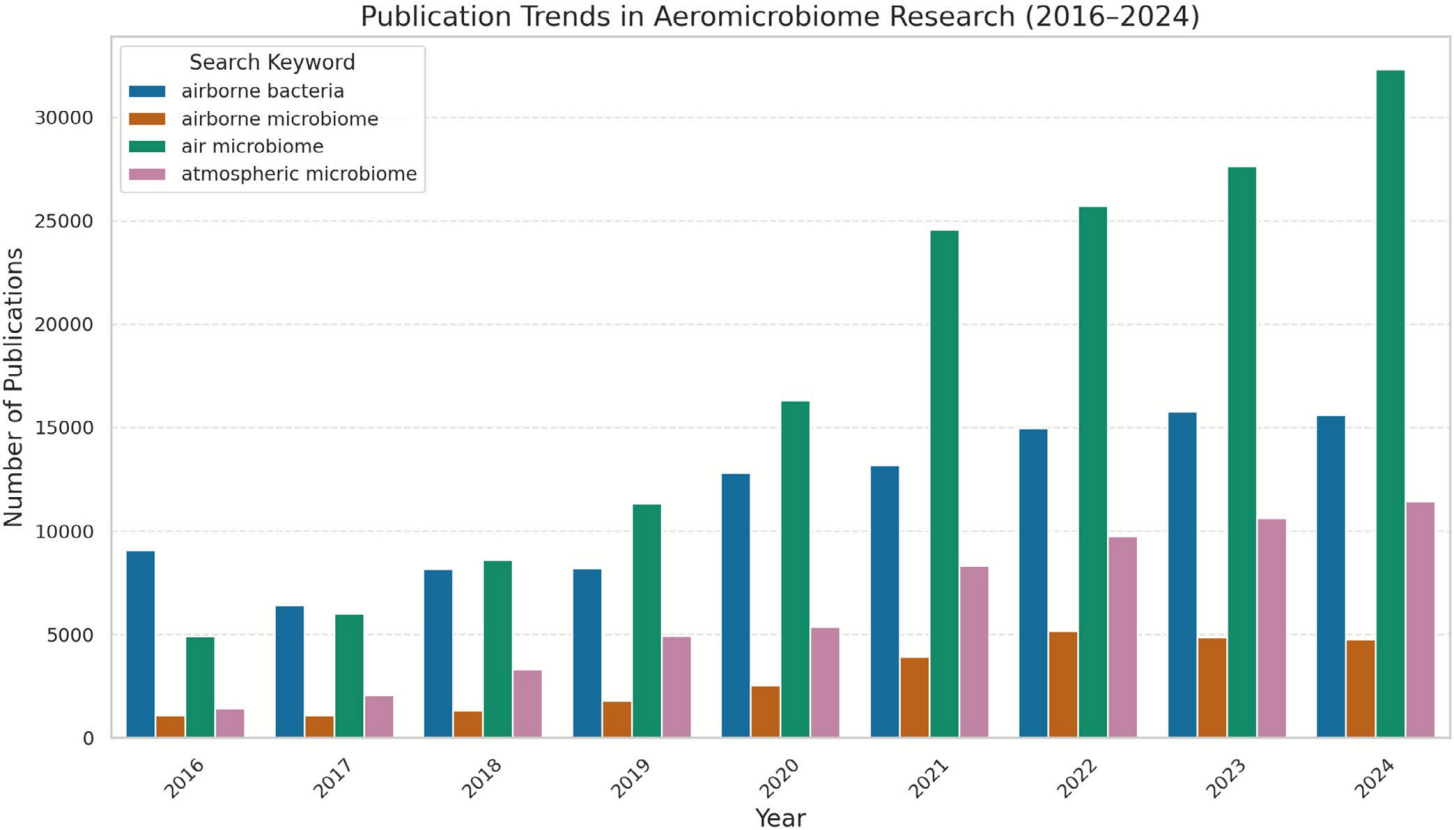
Publication trends in aeromicrobiome research from 2016 to 2024, based on Dimensions keyword searches. The bar plot shows annual publication counts for four commonly used search terms: ‘airborne bacteria’, ‘airborne microbiome’, ‘air microbiome’, and ‘atmospheric microbiome’.

The atmosphere is increasingly recognised as a dynamic microbial habitat. Although interest in the aeromicrobiome has grown substantially over the past decade (Figure 1), our understanding of it remains limited in scope and depth compared with aquatic and terrestrial systems. Most studies rely on 16S rRNA gene amplicon sequencing, which restricts taxonomic resolution and functional interpretation (Zhao et al. 2022; Tignat‐Perrier et al. 2019; Archer et al. 2022). Evidence for in situ activity and genomic diversity is scarce, and comparative or experimental studies under aerosol conditions are rare. Low biomass, methodological challenges in sampling and the need for strict contamination control are some factors that constrain progress toward deeper multi‐omics analyses (Bowers et al. 2013; Šantl‐Temkiv et al. 2022).

The atmosphere subjects microbes to extreme selective pressures, including ultraviolet radiation, desiccation, oxidative stress, low pH and nutrient limitation (Smith et al. 2011; Chen et al. 2020). It is believed that most airborne bacteria die shortly after aerosolisation, but some possess physiological traits that enable survival during atmospheric residence (Santl‐Temkiv et al. 2017). Available evidence indicates that viability often declines within minutes to hours under sunlit, low‐humidity conditions (Tong and Lighthart 2000; Després et al. 2012). Despite their low local abundance, airborne bacteria collectively form a dynamic and globally distributed pool seeded by terrestrial and aquatic sources (Zhao et al. 2022). Interest in these communities has accelerated markedly over the past decade, with publication rates increasing, particularly since 2020 (Figure 1).

Airborne bacterial abundance varies widely, typically ranging from 10^2^ to 10^7^ cells per metre cube of air depending on altitude, location and weather conditions (Bowers et al. 2011; Tignat‐Perrier et al. 2020). Although this density is low relative to soil or aquatic environments, the global troposphere is estimated to contain approximately 10^20^ bacterial cells at any time (Whitman et al. 1998). This biomass is continually replenished through emissions from soil, vegetation, water bodies and human activity. Amplicon‐based surveys reveal that airborne bacterial communities are not randomly assembled but exhibit global patterns in diversity and composition (Zhao et al. 2022). Taxonomic richness is high at the global scale, and certain genera are consistently detected across continents and seasons. At the same time, community composition varies across regions and site types (Pollegioni et al. 2023). Recent multi‐site sampling in the central Mediterranean showed spatial structuring in airborne bacterial and fungal assemblages, reflecting regional and environmental influences (Fragola et al. 2025). Comparable variation linked to underlying land use, such as differences between urban and suburban air, has been documented in other regions (Stewart et al. 2020), and large‐scale transect sampling on the East Antarctic Plateau further demonstrates biogeographic structuring driven by wind trajectories (Parro et al. 2025).

While many airborne microbes may be inactive or short‐lived in the aerosol phase, a subset of microbes survive and may influence atmospheric processes. Some bacteria, first exemplified by *Pseudomonas syringae* (Maki et al. 1974), produce ice‐nucleating proteins (INPs) that catalyse ice formation at relatively high subzero temperatures and can promote cloud formation and precipitation. Biological ice nucleation and *P. syringae* have been detected in atmospheric aerosols and clouds (Christner et al. 2008). Others metabolise trace gases or interact with organic aerosols, linking microbial activity to atmospheric chemistry (Ervens et al. 2025). These potential functions highlight the need to investigate the physiological and ecological traits that support microbial survival and activity in the air.

In this mini‐review, we focus on bacterial taxa that are frequently detected and abundant in airborne microbial surveys. Although these genera are widespread in soil, water and plant‐associated environments, their repeated detection in air likely reflects ecological filtering under atmospheric stressors. The atmosphere receives continuous microbial inputs from surrounding surfaces, yet only a subset of broadly stress‐tolerant taxa persist long enough to be consistently observed. Their prevalence probably reflects general resilience traits such as desiccation tolerance and oxidative stress resistance. At present, it is unclear whether any lineages possess adaptations that are specific to the airborne environment, although future comparative genomic and experimental work may clarify this. Most available datasets come from the lower troposphere, where sampling is feasible and atmospheric residence times are relevant for microbial survival, so this review mainly considers this atmospheric layer.

Although archaea are also present in air, most airborne community surveys have used bacterial‐targeted 16S rRNA gene amplicon workflows (commonly targeting the V3‐V4 region) or earlier “universal” primers with limited archaeal coverage (Tignat‐Perrier et al. 2019; Zhao et al. 2022; Archer et al. 2022). As a result, archaeal diversity is often underrepresented in airborne amplicon datasets. Studies that include archaeal‐resolving sequencing consistently report airborne assemblages dominated by Euryarchaeota and Thaumarchaeota, with lower abundances of Crenarchaeota and Woesearchaeota (Fröhlich‐Nowoisky et al. 2014; Wehking et al. 2018; Cáliz et al. 2018). Their sources were traced mainly to soil and dust, and abundant classes such as *Methanobacteria, Methanomicrobia* and *Thaumarchaeota* indicate potential links with methanogenesis and ammonia oxidation (Niu et al. 2021). Given the strictly anaerobic lifestyles of methanogens which were found to be abundant, it is however unlikely that they are active or even surviving in the atmosphere. Archaea represent a minor fraction of airborne microbial communities, typically comprising less than 1% of total 16S rRNA gene reads in mixed‐domain surveys (Niu et al. 2021; Cáliz et al. 2018; Fröhlich‐Nowoisky et al. 2014). Given this, most of the available evidence on diversity, survival and function therefore pertains to bacteria, and the remainder of this review focuses on ecological characteristics that shape airborne bacterial communities, emphasising survival strategies and functional adaptations to the atmospheric environment. Drawing on recent global surveys, we highlight frequently detected and abundant bacterial taxa and explore physiological traits that may support their survival in air. We also review emerging evidence of metabolic activity during atmospheric residence and consider how functionally specialised bacteria may contribute to atmospheric processes.

## 2 Selective Pressures in the Atmosphere and Survival Strategies

**FIGURE 2:**
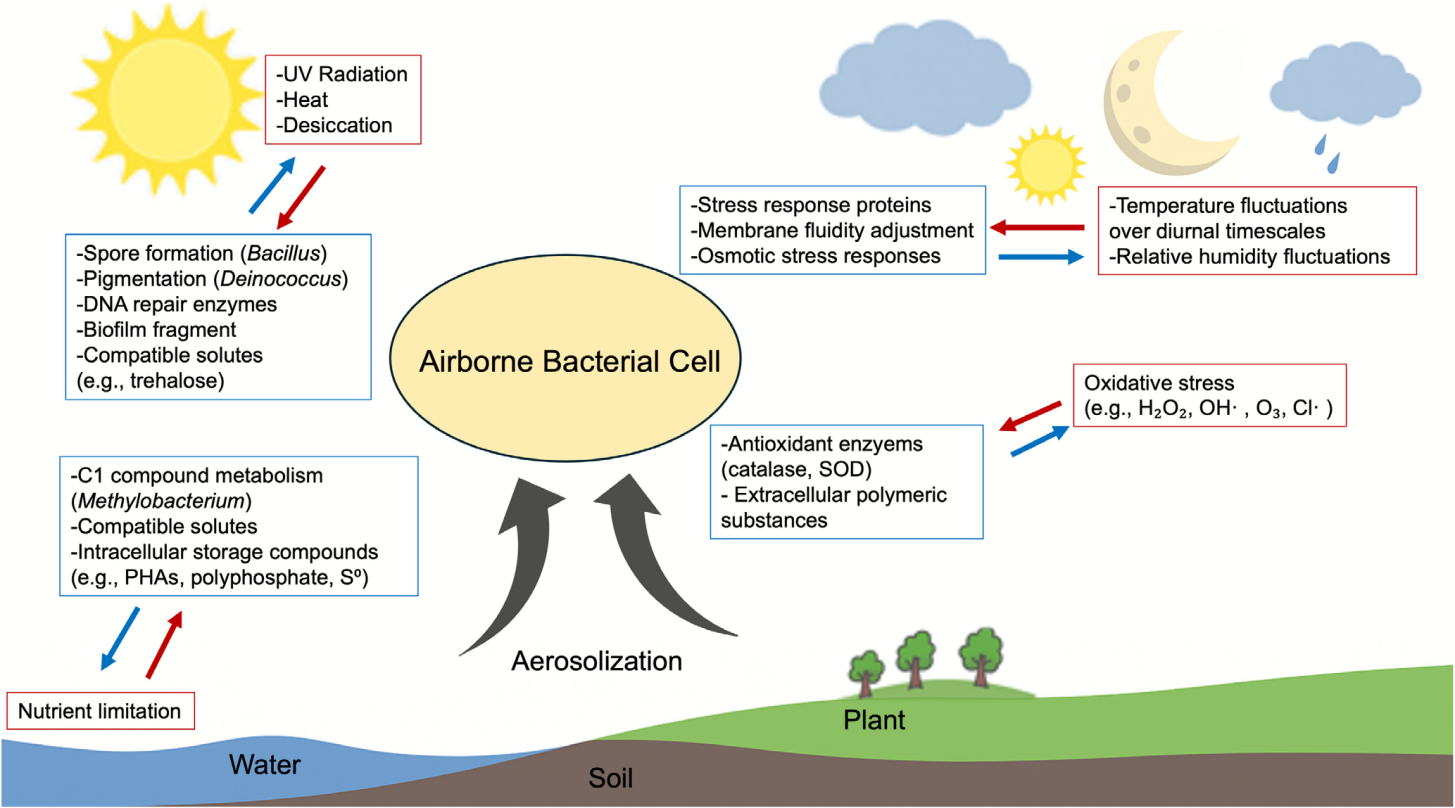
Conceptual schematic illustrating selective pressures in the atmosphere and the survival strategies of airborne bacteria. Processes such as aerosolisation transport cells from terrestrial and aquatic environments into the air, where they encounter ultraviolet radiation, extreme and rapidly fluctuating temperatures, low water activity, exposure to oxidative stressors, and nutrient limitation. Arrows indicate directional relationships: red for stressors and blue for microbial responses.

The atmosphere presents a uniquely hostile environment for bacteria, imposing multiple abiotic stressors that differ substantially from conditions on land or in water. Upon aerosolisation, microbes are exposed to high levels of ultraviolet (UV) radiation, oxidising agents including hydroxyl radicals, ozone, oxygen and chlorine atoms, low humidity, low water activity and low pH, large fluctuations in temperatures over short timescales and severe nutrient limitation (Madronich et al. 2018; Pell et al. 1997; Gusareva et al. 2019). These combined stressors contribute to high mortality and restrict survival to physiologically resilient taxa. Viability studies using membrane integrity staining show that most airborne bacteria lose viability quickly under atmospheric conditions. For example, staining of high‐altitude air samples revealed that only 3%–7% of cells had intact membranes, with nighttime samples showing slightly higher proportions likely due to reduced UV exposure (Santl‐Temkiv et al. 2017). Laboratory experiments in an atmospheric simulation chamber confirm this pattern of rapid viability loss: aerosolised *Escherichia coli* exhibit a median viable lifetime of just 32 min under baseline conditions of ambient temperature (~20°C) and moderate humidity (60% RH), in the absence of UV radiation and oxidising agents (Vernocchi et al. 2023). These experiments likely underestimate the full lethality of real atmospheric conditions, where UV and oxidising agents can cause DNA and cellular damage and lower humidity accelerates cellular water loss. These results highlight the severity of airborne stress and the selective advantage of microbial traits that promote survival (Figure 2).

Despite extreme stressors that result in high mortality rates, certain bacterial taxa are consistently detected in air across diverse atmospheric environments globally (Zhao et al. 2022; Tignat‐Perrier et al. 2019). Their recurrent presence implies the existence of conserved traits that improve survival under airborne stress. In the following, we outline key environmental pressures encountered in the atmosphere and the physiological strategies that bacteria employ to survive during atmospheric residence (Figure 2).

1. *Spore formation and dormancy*: Spore formation is often suggested as a survival strategy that may contribute to microbial survival in the atmosphere (Maus et al. 2001; Smets et al. 2016). Spores produced by genera such as *Bacillus* are indeed resistant to desiccation, UV radiation and heat due to protective features like the spore coat, dipicolinic acid and small acid‐soluble proteins (Nicholson et al. 2000; Dean et al. 2022). However, sporulation is a slow process that typically takes 8 to 10 h and is triggered by nutrient depletion under laboratory conditions (Mckenney et al. 2013). Given that vegetative cells typically lose viability within minutes to hours after aerosolisation, it is unlikely that sporulation occurs after aerosolisation. Germination is also improbable during atmospheric residence because it requires specific environmental cues such as moisture and nutrients, which are not abundant in aerosols. Germination may be possible within cloud droplets, which transiently provide hydrated microenvironments with dissolved solutes, though this has not thus far been observed or demonstrated. Despite these constraints, members of the spore‐forming genus *Bacillus* are among the most frequently detected and abundant genera in airborne microbial surveys (Zhao et al. 2022). This suggests that pre‐formed spores from terrestrial sources may be passively aerosolised and survive longer than vegetative cells due to their inherent resilience. Therefore, spore formation may enhance airborne survival, but not as an active physiological response to atmospheric stress.
2. *Pigmentation*: Pigmentation enhances microbial tolerance to UV radiation. Carotenoids and melanin‐like pigments present in genera such as *Deinococcus* and *Micrococcus* provide antioxidant functions and absorb light in the UV region of the spectrum, protecting cellular structures from photodamage (Farci et al. 2016; Slade and Radman 2011). These pigmented taxa are frequently isolated from air (Tong and Lighthart 1997). Their pigmentation is consistent with protection from UV/solar stress and may offer a selective advantage in atmospheric environments.
3. *Oxidative stress resistance*: The atmosphere is a highly oxidising environment (Thompson et al. 2024). Major oxidants such as ozone are substantially depleted near the ground due to rapid dry deposition and surface chemical reactions. Airborne measurements show ozone concentrations of ~70 ppb aloft compared with ~25 ppb at the surface (Berkowitz and Shaw 1997), suggesting strong oxidant attenuation in the near‐surface boundary layer. Airborne microbes are therefore exposed to a range of oxidising agents, including reactive oxygen species (ROS) such as ozone and hydroxyl radicals, as well as oxidants like molecular oxygen and chlorine. These compounds can damage DNA, proteins and membranes, leading to loss of viability. To counteract this, bacteria rely on antioxidant defence systems, including a suite of enzymes including catalases, peroxidases and superoxide dismutases, which detoxify ROS and help prevent oxidative damage to essential cellular components (Vaïtilingom et al. 2013; Oswin et al. 2023). Functional gene expression studies indicate that stress‐response pathways including antioxidant defences are active in cloud water and clear‐air aerosols (i.e., non‐cloud) (Amato et al. 2017; Péguilhan et al. 2025). In clear‐air aerosol samples, transcripts linked to oxidative stress and DNA damage are particularly evident (Péguilhan et al. 2025). Causal evidence that these defences enhance airborne bacterial survival remain limited, but an *E. coli* *sodA* mutant shows reduced viability under aerosol conditions, consistent with a protective role for superoxide dismutase (Oswin et al. 2023). Complementary laboratory studies under oxygen‐rich conditions show that mutants lacking antioxidant enzymes exhibit oxidative damage and reduced growth, further highlighting the importance of antioxidant defences in coping with oxidative stress (Oh et al. 2015). In addition to endogenous ROS formation, photochemical aging of metal‐containing dust and secondary organic aerosols can generate particle‐bound oxidants (Kilchhofer et al. 2024). These exogenous oxidants could exacerbate membrane peroxidation and DNA damage in airborne microorganisms.
4. *Desiccation tolerance and DNA protection*: Microbes suspended in air often encounter low water activity that causes osmotic stress, disrupts membrane integrity and damages nucleic acids. Desiccation can cause DNA strand breaks and oxidative lesions, particularly during rehydration when intracellular ROS levels increase sharply (Ball 2008; Romero‐Perez et al. 2023). Some bacteria accumulate compatible solutes such as trehalose, ectoine and glycine betaine to preserve protein structure and stabilise membranes in low water activity environments (Delort et al. 2017; Thomas et al. 2025). These compounds may also serve as energy and carbon sources under nutrient‐limited conditions (Thomas et al. 2025). Additional strategies include surface modifications that enhance desiccation resistance (Wang et al. 2025), as well as the production of stress‐response proteins, DNA repair enzymes and extracellular polymeric substances (EPS), which help retain moisture by slowing water loss (Roberson and Firestone 1992). EPS can also trap dissolved compounds such as nutrients (Costa et al. 2018; Flemming and Wingender 2010), thereby buffering cells against water loss and nutrient limitation. Complementing EPS‐based protection, recent proteomic and lipidomic analyses show that airborne *Escherichia coli* up‐regulate lipid biosynthesis during aerosolisation and form lipid‐containing droplets that repair outer‐membrane lesions caused by airflow and desiccation, thereby maintaining envelope integrity (Smith et al. 2025).
5. *pH extremes and membrane homeostasis*: Atmospheric particles exhibit a wide range of acidity. Field observations and models show that fine aerosols and cloud water often reach low pH because their acidity is controlled by condensable acids such as HNO_3_ and H_2_SO_4_, partial buffering by ammonia, and dynamic gas–particle exchange (Pye et al. 2020; Shah et al. 2020). Particle pH can shift rapidly as it equilibrates with surrounding air, and hour‐to‐hour changes have been observed in ambient measurements (Shi et al. 2019). Experimental simulations of cloud water show that bacterial survival and metabolism are sensitive to pH within the range of 3–6, with viability decreasing under more acidic conditions, indicating that acidic conditions impose physiological stress on microbial cells in the atmosphere (Liu, Lim, et al. 2022). These findings suggest that microorganisms aerosolised in the atmosphere may rely on pH homeostasis and acid‐tolerance mechanisms such as proton‐pumping ATPases, stress‐response proteins and extracellular buffering (Liu, Lim, et al. 2022). In addition, membrane transporters and mechanosensitive channels likely contribute to pressure and osmotic homeostasis during rapid humidity and airflow fluctuations, supporting microbial survival under dynamic atmospheric conditions (Ramirez et al. 2023; Blount and Iscla 2020).
6. *Nutrient limitation and resource storage*: Atmospheric microbes often experience prolonged nutrient limitation, which may constrain their metabolic activity and viability. As a potential adaptation to such oligotrophic conditions, some bacteria accumulate intracellular storage compounds that serve as reserves of carbon, energy, or essential nutrients. These include polyhydroxyalkanoates (PHAs) (Kadouri et al. 2005), polyphosphate granules (Jendrossek 2021) and elemental sulphur globules (Benisch et al. 2024), which have been shown to support microbial survival under starvation in non‐airborne systems. While not specific to atmospheric exposure, such traits may confer a physiological advantage during atmospheric residence by enabling cells to carry out maintenance metabolism in the absence of external nutrients.

## 3 Physiological Traits of Prevalent Airborne Bacteria That May Enhance Survival

Despite the harsh selective pressures of the atmosphere, a global meta‐analysis of airborne bacterial communities identified a consistent set of dominant bacterial taxa across 11 countries (Zhao et al. 2022). Genera such as *Methylobacterium*, *Sphingomonas*, *Bacillus* and *Hymenobacter* were not only frequently observed, but also ranked among the most abundant globally across diverse geographic regions and land‐use types (Zhao et al. 2022). While local community composition varied, the recurrent dominance and high relative abundance of this limited group suggest that the atmosphere acts as an environmental filter, favouring hardy and metabolically flexible lineages adapted to aerosolisation and atmospheric suspension (Behzad et al. 2015; Burrows et al. 2013).

To identify traits that may underlie atmospheric survival, we summarised experimentally supported survival features of the 10 most abundant and frequently detected airborne genera reported by Zhao et al. (2022) (Table 1). These genera span four bacterial phyla: Pseudomonadota, Bacillota, Bacteroidota and Actinomycetota, suggesting that atmospheric survival traits are phylogenetically widespread. The prevalence of these taxa is further supported by other large‐scale airborne surveys. For example, *Methylobacterium* was consistently among the most abundant airborne genera in a recent survey across multiple campus environments in China, reaching up to 12% relative abundance overall (Zhang, Liu, et al. 2024). *Sphingomonas* and *Bacillus* were also commonly detected across diverse global sampling sites, including urban and high‐altitude locations, and ranked among the top genera in both relative abundance and occurrence (Tignat‐Perrier et al. 2019; Woo and Yamamoto 2020). These patterns, observed across studies with diverse geographic and environmental contexts, underscore the widespread prevalence of these genera in atmospheric bacterial communities. For in‐depth discussion, we focus on four genera (*Methylobacterium*, *Sphingomonas*, *Bacillus* and *Hymenobacter*), which were among the most abundant and consistently detected taxa across sampling locations and land‐use types. These examples illustrate diverse survival strategies and provide insight into trait‐based filtering by the atmospheric environment.

**TABLE 1:** Potential survival traits of the 10 most abundant and frequently detected airborne bacterial genera, listed in order of overall abundance based on global airborne microbial surveys (Zhao et al. 2022).

Genus (phylum)	Potential survival traits	References
*Methylobacterium* (Pseudomonadota)	Desiccation tolerance; C1 metabolism (methanol utilisation); photoheterotrophy	Yano et al. (2013); Vuilleumier et al. (2009); Zhang, Liu, et al. (2024); Omer et al. (2004)
*Sphingomonas* (Pseudomonadota)	Stress resistance (ionising radiation); biofilm formation; C1 metabolism; photoheterotrophy; adaptability	Ragon et al. (2011); Chan et al. (2023); Salka et al. (2014); Amato et al. (2023)
*Bacillus* (Bacillota)	Endospore formation; resistance to desiccation, heat, and radiation; antimicrobial compound production	Setlow (2006); Wang et al. (2014); Maldonado et al. (2009)
*Hymenobacter* (Bacteroidota)	Desiccation tolerance; adaptation to oligotrophic conditions; metabolic versatility	Buczolits et al. (2002); Cha et al. (2020); Tanner et al. (2020)
*Turicibacter* (Bacillota)	Host‐associated anti‐inflammatory traits; niche adaptation	Terzo et al. (2020); Rausch et al. (2015); Lynch et al. (2023)
*Thermoactinomyces* (Bacillota)	Thermotolerance; thermostable enzymes; bioactive compounds	Song et al. (2001); Verma et al. (2016); Wu et al. (2020); Hou et al. (2024)
*Acinetobacter* (Pseudomonadota)	Biofilm formation; desiccation resistance; environmental adaptability	Nait Chabane et al. (2014); Lopez‐Gigosos et al. (2019); Zhao et al. (2023)
*Pseudomonas* (Pseudomonadota)	Desiccation resistance; biofilm formation; metabolic versatility; ice nucleation	Aladejana et al. (2021); Amato et al. (2007); Silby et al. (2011); Fahlgren et al. (2011)
*Rubellimicrobium* (Pseudomonadota)	Aerobic metabolism; pigmented UV protection; metabolic versatility; biogeochemical cycling	Weon et al. (2009); Fiebig et al. (2013); Cao et al. (2010); Han et al. (2023)
*Geodermatophilus* (Actinomycetota)	Resistance to oxidative stress and ionising radiation; Desiccation and UV resistance; biofilm formation; organic degradation	Gtari et al. (2012); Xie and Pathom‐Aree (2021); Tignat‐Perrier et al. (2020)

### 3.1 Methylobacterium

The frequent detection of *Methylobacterium* in air is likely supported by traits such as desiccation tolerance, C1 metabolism and stress protection mechanisms. This genus is well adapted to low‐moisture environments and biofilm formation contributes to maintaining cellular integrity under low water activity conditions (Yano et al. 2013). Although biofilms are unlikely to form in the atmosphere, fragments of previously formed biofilms or EPS‐embedded cell aggregates are commonly aerosolised from surfaces and dust, and may provide transient protection during suspension. A key feature of *Methylobacterium* is its ability to utilise C1 compounds like methanol, which is commonly emitted by plants and present in the phyllosphere and potentially in aerosols (Vuilleumier et al. 2009). Genes involved in methanol metabolism, including those regulating purine and amino acid biosynthesis, have been shown to enhance colonisation of plant surfaces by supporting efficient resource use under nutrient‐limited conditions (Zhang, Zhou, et al. 2024). These same metabolic pathways may incidentally support airborne survival by allowing cells to utilise trace organic compounds encountered during atmospheric residence. Some *Methylobacterium* strains produce carotenoid pigments, which contribute to oxidative and light‐induced stress resistance on exposed surfaces (Omer et al. 2004). These pigments likely enhance survival under atmospheric conditions by protecting cells from UV and ROS.

### 3.2 Sphingomonas

The frequent detection of *Sphingomonas* in air may reflect its stress tolerance, metabolic versatility and surface‐associated traits that enhance desiccation resistance. This genus can oxidise a wide range of organic compounds, including aromatic hydrocarbons, alcohols and C1 compounds, a feature that may provide a competitive advantage under nutrient‐limited atmospheric conditions (Stolz 2009; Fredrickson et al. 1995). Species in this genus have been detected in biofilms that are resistant to high levels of ionising radiation (Ragon et al. 2011). Their pigmentation, often involving carotenoids, may contribute to protection against oxidative stress and radiation damage by scavenging free radicals and shielding cellular components (Stolz 2009). *Sphingomonas* spp. frequently form biofilms on environmental surfaces, and cells embedded in these extracellular matrices exhibit enhanced resistance to low water activity and UV‐associated stress (Yin et al. 2019). Such aerosolised biofilm fragments or EPS‐embedded aggregates from plant or dust may enhance the atmospheric survival of embedded cells via matrix protection. Some *Sphingomonas* strains, including strain AAP5, have been shown experimentally to perform aerobic anoxygenic photoheterotrophy, using bacteriochlorophyll‐a to harvest light while relying on organic compounds for carbon and energy (Kopejtka et al. 2021). This light‐harvesting capability may be advantageous in light‐exposed airborne particles where nutrients are limited. In addition, certain strains degrade complex aromatic compounds like humic substances, expanding their metabolic repertoire under variable environmental conditions (Salka et al. 2014). Several *Sphingomonas* spp. exhibit growth at moderately acidic pH (as low as pH 4–5) and are abundant in acidic soils (pH 2–4) (Han et al. 2025), suggesting a general tolerance to low‐pH environments that may incidentally support persistence in acidic atmospheric particles (Kawasaki 2010; Han et al. 2025). Genomic and comparative analyses show that *Sphingomonas* harbour genes associated with oxidative stress resistance, pollutant degradation and mobile genetic elements (Liu, Cui, et al. 2022; Lombardino et al. 2022; Zhao et al. 2017). These traits likely support adaptation to oxidative, nutrient‐limited and variable atmospheric conditions.

### 3.3 Bacillus

*Bacillus* is frequently detected and relatively abundant in air, which may be related to its ability to form endospores. Sporulation allows cells to transition into a dormant state that is highly resistant to desiccation, UV radiation, heat and nutrient deprivation (Nicholson et al. 2000; Setlow 2006; Wang et al. 2014). These spores can remain viable for extended periods, making them well suited for atmospheric survival. However, sporulation is likely not a direct response to atmospheric stress, as it requires nutrient depletion and takes several hours to complete (Mckenney et al. 2013). It is therefore unlikely to successfully occur after aerosolisation. Instead, spores are likely formed in terrestrial or aquatic environments and are then passively aerosolised. In addition to spore‐mediated protection, several *Bacillus* species show substantial tolerance to acidic conditions, with vegetative cells surviving at pH 4–5 and acid‐adapted strains persisting in even more acidic environments (Mols and Abee 2011; Wilks et al. 2009; Chen et al. 2009). Such acid resilience may incidentally support survival in acidic atmospheric particles. Many *Bacillus* spp. form biofilms that retain moisture and stabilise cells under fluctuating humidity and mechanical stress (Vlamakis et al. 2013). These additional traits, while not specific to the airborne phase, may incidentally enhance survival in the atmosphere.

### 3.4 Hymenobacter

*Hymenobacter* is another genus frequently detected in air that exhibits traits that could support survival under atmospheric stress, including desiccation resistance, oligotrophic growth and radiation tolerance. Several strains (e.g., *Hymenobacter roseosalivarius* ) remain viable after prolonged exposure to low water activity, indicating a high level of tolerance to low‐humidity conditions (Buczolits et al. 2002). *Hymenobacter* spp. have also been found on surfaces characterised by intense radiation and low water availability, such as photovoltaic panels, often co‐occurring with other extremotolerant taxa (Tanner et al. 2020). These strains frequently produce protective pigments such as carotenoids, which function as antioxidants and may contribute to membrane stabilisation and oxidative stress resistance (Klassen and Foght 2008). The genus is commonly associated with nutrient‐poor habitats such as tree bark, rock surfaces and desert soils (Cha et al. 2020; Lai et al. 2024). Some strains can hydrolyse substrates, including gelatin, starch, xylan and Tween compounds, supporting the view that *Hymenobacter* are metabolically flexible and can utilise diverse organic compounds under oligotrophic conditions (Buczolits and Busse 2015). Such adaptability is relevant to the atmosphere, where organic substrates are present in limited concentrations.

Across these examples, the most frequently detected airborne bacteria globally appear to share key survival traits that likely underlie their recurrent presence across diverse environments. Common features include desiccation tolerance, metabolic flexibility and diverse mechanisms that confer resistance to environmental stressors like UV radiation and oxidative damage. Many airborne taxa identified in clouds are organotrophs, capable of utilising a wide range of carbon substrates, including organic C1 compounds such as methanol and formaldehyde (Husárová et al. 2011; Amato et al. 2017). For example, *Methylobacterium* exhibits metabolic versatility, using both C1 compounds like methanol and multi‐carbon organics as energy sources. Recent experiments also suggest that some *Methylobacterium* strains isolated from cloud water exhibit facultative photoheterotrophy. These bacteria produce bacteriochlorophyll and show extended survival under light exposure. However, they do not fix CO_2_ and rely on organic substrates like methanol for growth, indicating that light supports but does not replace heterotrophic metabolism (Mathonat et al. 2025). Similar light‐assisted survival has been demonstrated in some *Sphingomonas* strains (Kopejtka et al. 2021). Additionally, recent studies show that some microbes can use trace gases such as carbon monoxide and hydrogen to survive in nutrient‐poor terrestrial and marine environments. Atmospheric hydrogen gas concentrations can drive ATP synthesis via a membrane‐bound hydrogenase (Soom et al. 2025), and atmospheric CO can be oxidised through quinone‐linked electron transfer into the respiratory chain, providing a direct energy source for maintenance metabolism (Kropp et al. 2025). Although such processes have not yet been directly confirmed in airborne microbes, these findings provide evidence that atmospheric concentrations of certain substrates could support survival and maintenance, if not growth, during atmospheric residence (Greening and Grinter 2022; Cordero et al. 2019). Traits such as spore formation and biofilm development further support survival under the fluctuating and nutrient‐poor conditions of the atmosphere. Many of these traits likely evolved in terrestrial or aquatic environments but may incidentally contribute to survival in the atmosphere. It is important to note, however, that while these traits support survival during atmospheric residence, they do not necessarily indicate metabolic activity in the atmosphere on their own. Whether airborne microbes remain metabolically active during atmospheric suspension remains an open question–one that recent molecular and experimental studies have begun to address.

## 4 Evidence for Microbial Activity in the Atmosphere

Multiple lines of evidence indicate that a fraction of airborne microbes remain viable and metabolically active during atmospheric residence, especially under moist conditions such as within clouds (Agranovski et al. 2002; Péguilhan et al. 2025; Amato et al. 2017). Viability staining and culture recovery consistently detect living cells, particularly under high‐humidity or nighttime conditions when UV and desiccation stress are reduced (Santl‐Temkiv et al. 2017). These findings suggest that certain atmospheric conditions can transiently mitigate environmental stressors and promote microbial survival.

For instance, microbial ATP has been detected in cloud water, indicating that a substantial fraction of cells in clouds may be viable (Hu et al. 2017). Cloud droplets provide hydrated microenvironments with variable but typically acidic pH (approximately 3–6), moderate ion availability and limited oxidative stress, enabling microbes to engage in maintenance metabolism, as evidenced by transcriptomic data showing active gene expression in cloud communities (Amato et al. 2019; Péguilhan et al. 2025; Pye et al. 2020). Nevertheless, droplet‐scale modelling suggests that nutrient‐to‐cell ratios in individual droplets can be up to 10 orders of magnitude lower than in well‐studied oligotrophic surface waters, such as alpine lakes and open oceans (Ervens et al. 2025). Despite these limitations, *Pseudomonas syringae* retains ice‐nucleating activity after exposure to UV radiation, desiccation and low temperature, indicating that this function persists under simulated atmospheric stress (De Araujo et al. 2019). While direct expression of ice‐nucleation genes was not measured, transcriptomes of cloud samples revealed the expression of genes associated with stress response and maintenance metabolism in related organisms (Amato et al. 2019).

Evidence of microbial activity extends beyond cloud droplets. A comparative rRNA/rDNA analysis showed that certain rare airborne taxa, including members of the order Rhodospirillales, were overrepresented in the rRNA (active) fraction of air samples, suggesting that even low‐abundance microbes can contribute disproportionately to metabolic processes (Klein et al. 2016). Supporting this, laboratory studies demonstrate that *Sphingomonas aerolata* increases ribosome production when exposed to trace organic substrates in an aerosolised state, indicating active metabolic regulation rather than dormancy (Krumins et al. 2014).

Additional insight is provided by studies on methanotrophic bacteria that remain viable and functional in the air. Controlled chamber experiments indicated that aerosolised methanotrophs (e.g., *Methylocystis* and *Methylocaldum*) were capable of DNA replication when supplied with elevated methane concentrations under humid conditions (Dillon et al. 2022). In parallel, soil‐dwelling methanotrophs with extremely high affinity for methane have been identified, most notably *Methylocapsa gorgona*, which can grow using atmospheric air as its sole energy and carbon source by oxidising both methane and hydrogen (Schmider et al. 2024). While direct in situ evidence for gas oxidation in the aerosol phase is limited, these studies collectively support the concept that trace gas metabolism may allow certain microbes to survive and even grow in the low nutrient conditions present during atmospheric residence.

Recent culture‐independent studies of cloud water and ambient aerosols have revealed in situ activity in airborne bacterial communities, based on RNA‐focused profiling of field samples. For example, metatranscriptomic datasets report abundant transcripts related to ribosomal function, ATP synthesis and membrane transport, with active populations including genera such as *Sphingomonas* and *Pseudomonas* (Amato et al. 2017; Péguilhan et al. 2025). These findings suggest that dominant airborne taxa can maintain core physiological activity during suspension. Similarly, aerosolisation experiments with *E. coli* showed upregulation of stress response and protein synthesis genes (Ng et al. 2018), indicating active acclimation to atmospheric stressors rather than complete dormancy.

## 5 Conclusions and Future Directions

Recent advances suggest that the atmosphere is not merely a passive conduit for microbial dispersal, but a selective habitat where a subset of bacteria survive, remain active and may even grow. Taxonomic surveys consistently detect a limited set of stress‐tolerant genera across diverse environments, implying conserved survival strategies. These include desiccation tolerance, antioxidant defences and the ability to metabolise diverse scarce or unusual substrates such as C1 compounds and trace gases. However, the functional significance of airborne microbes remains underexplored, particularly regarding their in situ activity and ecological roles during atmospheric residence.

We assess the progress toward the three aims of this review, which are to synthesise airborne microbial diversity, the selective pressures that exist in the atmosphere, and the traits that support survival. For diversity, existing data show patterns and reveal consistently high frequency of detection of several bacterial taxa. However, due to the nature of the datasets, strain‐level resolution is limited and archaeal representation is poor. The key limitation here is that the underlying evidence is largely from global genus‐level 16S rRNA gene surveys, which restrict taxonomic and functional resolution. For selective pressures, the principal challenges that microbes face in air are fairly well described from atmospheric physics and chemistry, but only a few studies have directly tested these pressures through physiological or transcriptomic analyses of aerosolised cells. For survival traits, evidence for in situ activity has grown, but genome‐ and transcriptome‐level data remain sparse and uneven across sites and seasons. Many of the survival strategies we discuss are inferred from other environments or simplified systems and have not yet been demonstrated directly in aerosolised cells. Low biomass, short residence times and contamination risks continue to complicate links between detection, viability and process rates. To address these gaps, future studies should prioritise approaches that provide higher resolution (e.g., shotgun metagenomics) and differentiate active from dormant microbial populations in air, such as rRNA:rDNA comparisons and metatranscriptomic profiling across regions and seasons with concomitant collection of environmental variables. Experimental systems simulating atmospheric stressors (e.g., UV, desiccation, nutrient limitation) should be coupled with targeted culturing and gene expression studies to validate predicted survival traits. Isotope‐based methods (e.g., with ^13^C‐labelled methane, carbon monoxide or H_2_^18^O) could be used to test whether airborne microbes actively oxidise trace gases or exhibit activity while aerosolised, linking identity to function and providing direct evidence for microbial roles in atmospheric processes. Incorporating microbial activity into atmospheric models may reveal underappreciated contributions to biogeochemical cycling. Such integrative approaches will help reposition airborne bacteria from passive travellers to active participants in atmospheric processes.

## Author Contributions

**Jungsoo Park:** conceptualization, investigation, data curation, visualization, writing – original draft, writing – review and editing. **S. Jane Fowler:** writing – review and editing, supervision, funding acquisition, conceptualization.

## Funding

This work was supported by the Government of Canada's New Frontiers in Research Fund (NFRF). (NFRFE‐2022‐00163).

## Conflicts of Interest

The authors declare no conflicts of interest.

## Data Availability

All data discussed in this review were obtained from publicly available sources cited throughout the manuscript. No original data were generated or analysed for this work. The review synthesises findings from previously published studies and other documented materials, all of which are fully referenced.
